# Supplementary material for: Nanocrystal Encapsulation, Release and Application Based on pH-Sensitive Covalent Dynamic Hyperbranched Polymers
Source: Polymers (Basel). 2019 Nov 22;11(12):1926. doi: 10.3390/polym11121926 (PMC6960846; doi:10.3390/polym11121926)
Supplement: Supplementary file 1 [file polymers-11-01926-s001.pdf]

polymers

MDPI

Supplementary material:

Nanocrystal Encapsulation, Release and Application Based on pH-Sensitive Covalent Dynamic Hyperbranched Polymers

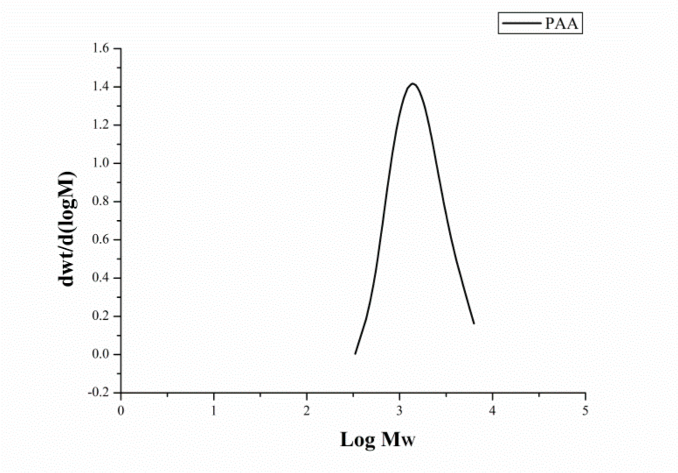

Figure S1. Evolution of molecular weight distribution of polyacrylic acid (PAA) obtained with gel permeation chromatography (GPC) using water as solvent.

Dist Name	Elution Volume (ml)	Retention Time (min)	Adjusted RT (min)	Mn	Mw	MP	Mz	Mz+1	Mz/Mw	Mz+1/Mw
	27.579	27.579	27.579	1188	1732	1375	2518	3401	1.45381	1.96363

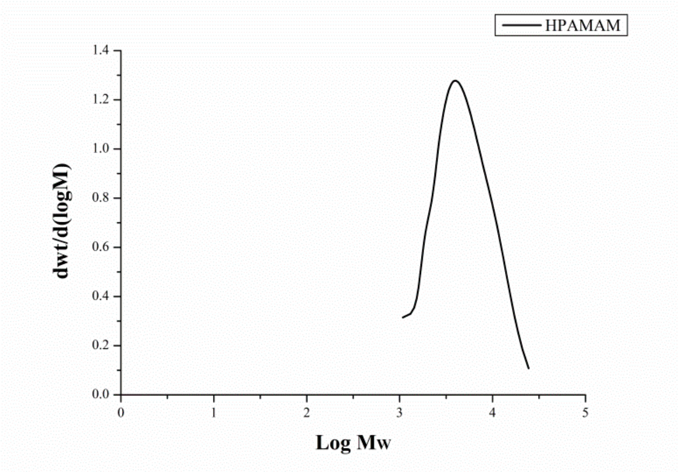

Figure S2. Evolution of molecular weight distribution of hyperbranched poly(amido amine)s (HPAMAM) obtained with GPC using water as solvent. RT: room temperature; Mn: number average molecular weight; Mw: weight average molecular weight; Mz: average molecular weight.

Dist Name	Elution Volume (ml)	Retention Time (min)	Adjusted RT (min)	Mn	Mw	MP	Mz	Mz+1	Mz/Mw	Mz+1/Mw
	25.324	25.324	25.324	3037	3848	3530	7726	11244	2.007796	2.922037

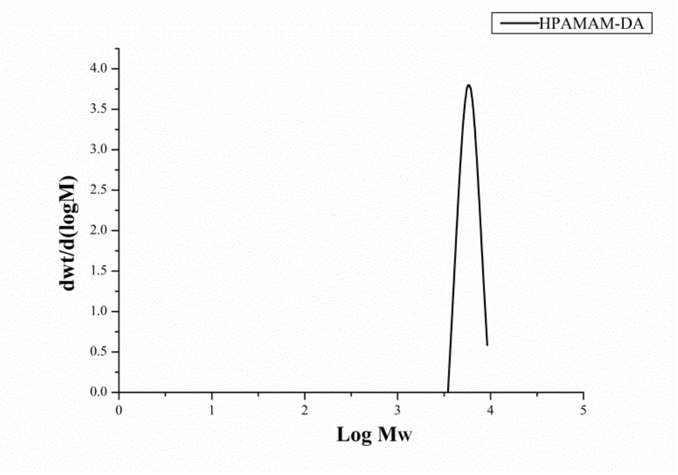

Figure S3. Evolution of molecular weight distribution of HPAMAM–DA obtained with GPC using chloroform as solvent.

Dist Name	Elution Volume (ml)	Retention Time (min)	Adjusted RT (min)	Mn	Mw	MP	Mz	Mz+1	Mz/Mw	Mz+1/Mw
	23.6	23.6	23.6	5694	5970	5956	6255	6540	1.047744	1.095528

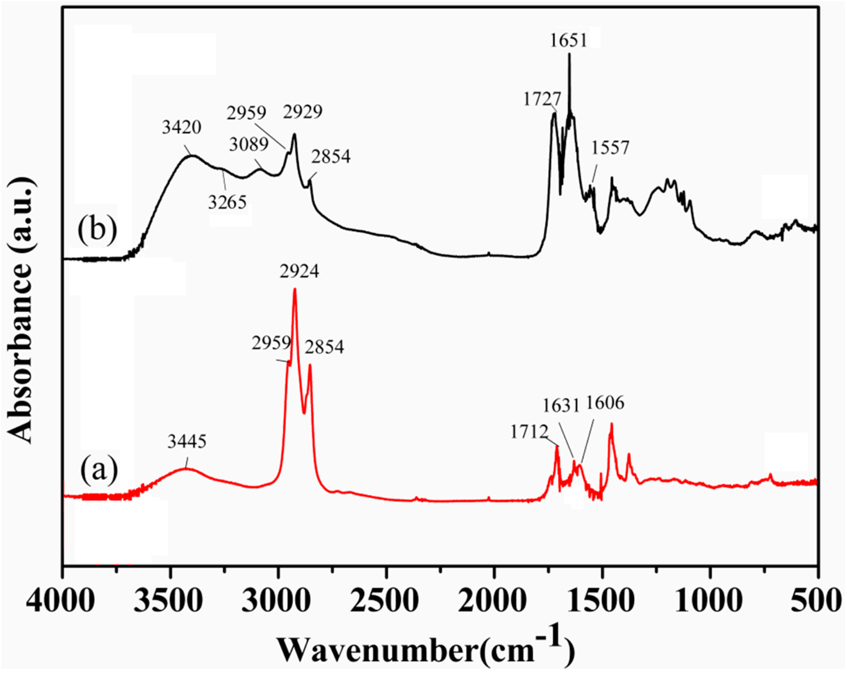

Figure S4. IR spectra of (**a**) the remained chloroform phase after imine hydrolysis of. HPAMAM–DA and (**b**) the final aqueous phase after imine hydrolysis of HPAMAM–DA.

(a) The remaining chloroform phase: IR (cm^{-1}), 3445 ($\nu_{as\ NH2}$), 2959 (ν_{CH3}), 2924($\nu_{as\ CH2}$), 2854 ($\nu_{s\ CH2}$), 1712 (ν_{CHO}), 1631 ($\nu_{C=N}$, δ_{NH2}), 1606 ($\nu_{C\text{-}N,}$ δ_{NH}).

(b) The final aqueous phase: IR (cm^{-1}), 3420 ($\nu_{as\ NH2}$), 3265 ($\nu_{s\ NH2}$), 3089 (ν_{NH}), 2959($\nu_{\ CH3}$), 2929($\nu_{as\ CH2}$), 2854 ($\nu_{s\ CH2}$), 1727 (ν_{CHO}), 1651 ($\nu_{C=N}$, δ_{NH2}), 1557 ($\nu_{C\text{-}N,}$ δ_{NH}).

The stretching vibration peaks at 2959 cm^{-1} (ν_{CH3}), 1712 cm^{-1} (ν_{CHO}, ν_{COOH}) in Figure S4b indicates the existence of protonated dodecyl aldehyde and PAA in the final aqueous phase after imine hydrolysis of HPAMAM–DA.

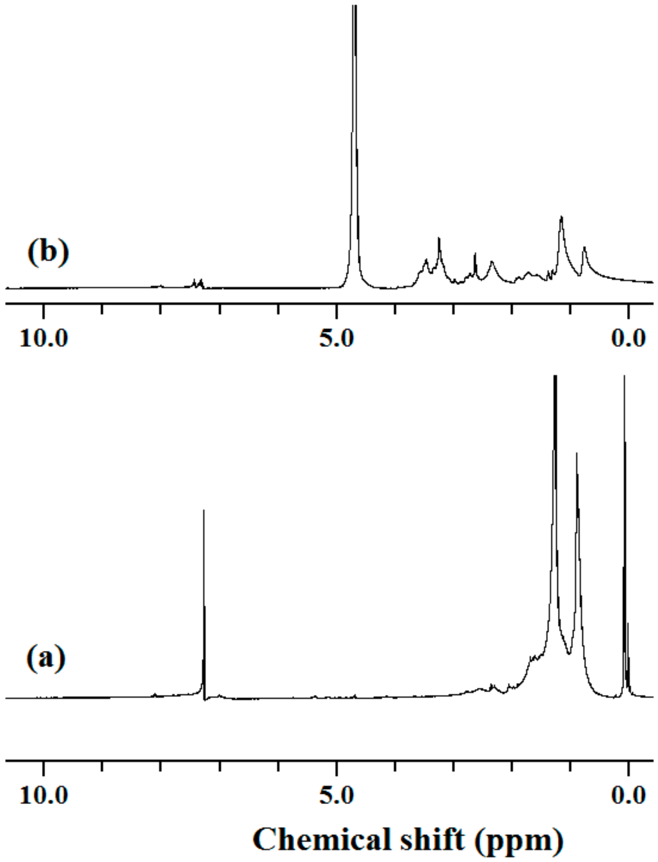

Figure S5. ^1H NMR spectra of (**a**) the remaining chloroform phase after imine hydrolysis of. HPAMAM–DA (400 MHz, in $CDCl_3$, 298 K) and (**b**) the final aqueous phase after imine hydrolysis of HPAMAM–DA (400 MHz, in D_2O, 298 K).

(a) The remaining chloroform phase after imine hydrolysis of HPAMAM-DA: ^1H NMR (400 MHz, $CDCl_3$, 298 K), 0.593–1.022 (CH_3), 1.043–1.897 ($(CH_2)_{10}$), 2.163–2.426 ($COCH_2$), 2.427–2.896 ($NHCH_2$, NH_2CH_2).

(b) The final aqueous phase after imine hydrolysis of HPAMAM–DA: ^1H NMR (400 MHz, D_2O, 298 K) δ: 0.609–1.043 (CH_3), 1.043–1.404 ($(CH_2)_{10}$, from protonated dodecyl aldehyde), 1.405–1.979 (CH_2, from PAA), 1.980–2.489 (NH_2, NH), 2.490–2.523($COCH_2$), 2.524–2.830 ($NHCH_2$, NH_2CH_2), 3.391–3.735 (NCH_2), 7.272–7.508 ($CONH$), 7.888–8.096 ($N{=}CH$).

The proton signals at 0.609–1.043 ppm (CH_3) and 1.043–1.404 ppm ($(CH_2)_{10}$) in the figure S5b indicates the exsitence of protonated dodecyl aldehyde.
